# A pan-*Theileria* FRET-qPCR survey for *Theileria* spp. in ruminants from nine provinces of China

**DOI:** 10.1186/1756-3305-7-413

**Published:** 2014-08-31

**Authors:** Yi Yang, Yongjiang Mao, Patrick Kelly, Zhangpin Yang, Lu Luan, Jilei Zhang, Jing Li, Heba S El-Mahallawy, Chengming Wang

**Affiliations:** Jiangsu Co-innovation Center for the Prevention and Control of Important Animal Infectious Diseases and Zoonoses, Yangzhou University College of Veterinary Medicine, Yangzhou, 225009 Jiangsu China; Yangzhou University College of Animal Science and Technology, Yangzhou, 225009 Jiangsu China; Ross University School of Veterinary Medicine, Basseterre, 00265 St. Kitts & Nevis; Faculty of Veterinary Medicine, Suez Canal University, Ismailia, 41225 Egypt

**Keywords:** *Theileria* spp, FRET-qPCR, Prevalence, Ruminants

## Abstract

**Background:**

*Theileria* spp. are tick transmitted protozoa that can infect large and small ruminants causing disease and economic losses. Diagnosis of infections is often challenging, as parasites can be difficult to detect and identify microscopically and serology is unreliable. While there are PCR assays which can identify certain *Theileria* spp., there is no one PCR that has been designed to identify all recognized species that occur in ruminants and which will greatly simplify the laboratory diagnoses of infections.

**Methods:**

Primers and probes for a genus-specific pan-*Theileria* FRET-qPCR were selected by comparing sequences of recognized *Theileria* spp*.* in GenBank and the test validated using reference organisms. The assay was also tested on whole blood samples from large and small ruminants from nine provinces in China.

**Results:**

The pan-*Theileria* FRET-qPCR detected all recognized species but none of the closely related protozoa. In whole blood samples from animals in China, *Theileria* spp. DNA was detected in 53.2% of the sheep tested (59/111), 44.4% of the goats (120/270) and 30.8% of the cattle (380/1,235). Water buffaloes (n = 29) were negative. Sequencing of some of the PCR products showed cattle in China were infected with *T. orientalis/T. sergenti/T. buffeli* group while *T. ovis* and *T. luwenshuni* were found in sheep and *T. luwenshuni* in goats. The prevalence of *Theileria* DNA was significantly higher in *Bos p. indicus* than in *Bos p. taurus* (77.7% vs. 18.3%) and copy numbers were also significantly higher (10^4.88^ vs. 10^3.00^*Theileria* 18S rRNA gene copies/per ml whole blood).

**Conclusions:**

The pan-*Theileria* FRET-qPCR can detect all recognized *Theileria* spp. of ruminants in a single reaction. Large and small ruminants in China are commonly infected with a variety of *Theileria* spp.

## Background

*Theileria* spp. are tick-transmitted, intracellular protozoan parasites infecting leukocytes and erythrocytes of wild and domestic large and small ruminants. Several *Theileria* spp., transmitted by ixodid ticks of the genera *Rhipicephalus*, *Hyalomma*, *Amblyomma* and *Haemaphysalis*, have been described in cattle, water buffaloes, sheep and goats in different geographical zones of the world [1–5]. Theileriosis is primarily limited to tropical and sub-tropical areas of the world, with infections mainly reported in Africa and the Middle East but also in southern Europe and northern Asia [6–11]. Infections by *Theileria* spp. can cause fever, anemia and hemoglobinuria and, in severe cases, death although many species are benign. Animals recovered from acute or primary infections usually remain persistently infected and may act as reservoirs of infecting ticks [12, 13].

While there have been numerous reports of theileriosis in various animal species in China since 1958 [14–27], many have been reported in Chinese and some were based on microscopic detection of parasites which can be difficult with low parasitemia and does not allow ready differentiation of species. Serological studies, although sensitive and easy to perform, are not specific as there is cross reactivity between *Theileria* spp. Although molecular studies have been performed, these have been to detect *Theileria* of specific domestic animal species, for example sheep and goats [27]. There have been no highly sensitive and specific molecular methods described which enable studies on various animals from widely divergent areas of China where different *Theileria* spp. might occur. To address this problem, we developed and validated a highly sensitive genus-specific *Theileria* FRET-qPCR that detects the recognized *Theileria* spp*.* of domestic animals and investigated the molecular prevalence of *Theileria* in cattle, water buffaloes, goats and sheep from nine provinces in China.

## Methods

### Animals and blood collection

Between 2007 and 2013, whole blood samples (around 6 ml) were collected in EDTA from apparently healthy cattle (n = 1,235), water buffaloes (29), goats (270) and sheep (111) from 9 provinces/municipality of China (Table 1). The *Bos primigenius* (*p*.) *taurus* studied (n = 975) were Holsteins, Simmentals, Bohai blacks, Luxis and Wannans while the *Bos. p. indicus* (n = 260) were the Yunlings, Minnans, and Leiqiongs (Table 1). The water buffaloes, goats and sheep in the study were bred in China and were indigenous breeds. Gender information was available for cattle from Yunnan province. After collection, the blood samples were frozen at -20°C and shipped on ice (over 2 days) to Yangzhou University where they were frozen at -80°C until thawed at room temperature for DNA extraction as described below. This study was reviewed and approved by the Institutional Animal Care and Use Committee of Yangzhou University and animal owners gave written permissions for blood collection.

**Table 1:** **Molecular prevalence of** ***Theileria*** **spp. in cattle, water buffalo, goat and sheep**

Animal species	Subspecies /breed		Province	City	Coordinate of city	***Theileria***positivity	
						positive /total	***%***
Cattle (n = 1235)	*Bos p. taurus*	Simmental	Inner Mongolia	Chifeng	42.17°N, 118.58°E	19/132	14.4%
		Bohai black	Shandong	Binzhou	37.22°N, 118.02°E	4/66	6.1%
		Luxi	Shandong	Jining	35.23°N, 116.33°E	40/40	100%
		Holstein	Jiangsu	Yancheng	33.22°N, 120.08°E	72/321	22.4%
		Holstein	Jiangsu	Yangzhou	32.23°N, 119.26°E	17/144	11.8%
		Holstein	Shanghai	Shanghai	31.14°N, 121.29°E	9/255	3.5%
		Wannan	Anhui	Wuhu	31.19°N, 118.22°E	17/17	100%
	*Bos p. indicus*	Yunling	Yunnan	Kunming	25.04°N, 102.42°E	124/161	77.0%
		Minnan	Fujian	Putian	24.26°N, 119.01°E	4/25	16.0%
		Leiqiong	Hainan	Haikou	20.02°N, 110.20°E	74/74	100%
Water buffalo (n = 29)	Haizi		Jiangsu	Yancheng	33.22°N, 120.08°E	0/29	0%
Goat (n = 270)	Xinjiang		Xinjiang	Urumqi	43.45°N, 87.36°E	4/98	4.1%
	Yangtse River Delta White		Jiangsu	Yangzhou	32.23°N, 119.26°E	116/172	67.4%
Sheep (n = 111)	Wuranke		Inner Mongolia	Xilingol	43.57°N, 116.03°E	36/72	50.0%
	Sishui Fur		Shandong	Jining	35.23°N, 116.33°E	23/39	59.0%

### DNA extraction

DNA was extracted from whole blood samples using a standard phenol-chloroform method previously described [28]. Two ml whole blood was used to extract DNA which was resuspended into 200 μl 1 × T_10_E_0.1_ buffer. The concentration of the extracted DNA was established with a Microscale Ultraviolet Spectrophotometer. Negative controls consisting of sterile molecular grade water were used to detect cross- contamination during DNA extraction and processing. The HMBS-based FRET-PCR was performed to verify if the extracted DNAs from blood samples were appropriate for molecular detection of tick-borne pathogens [29, 30].

### *Theileria*spp*.*FRET-qPCR

#### Primers and probes

**Figure 1:**
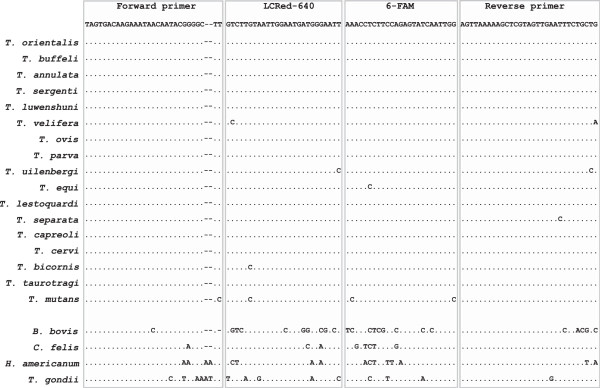
**Alignment of oligonucleotides for** ***Theileria*** **PCR used in this study.** Primers and probes are shown at the top of the boxes. Dots indicate nucleotides identical to primers and probes, and dashes denote absence of the nucleotide. The upstream primer is used as the demonstrated sequences without gaps while the two probes and downstream primer are used as antisense oligonucleotides. The designed oligonucleotides show minimum mismatching with *Theileria* spp. (0 mismatch with 11 species, 1 mismatch with 3 species, 2 mismatches with 2 species and 4 mismatches with 1 species), but significant numbers of mismatches with *Babesia bovis* (25 mismatches), *B. divergens* (23 mismatches), *B. bigemina* (22 mismatches), *Cytauxzoon felis* (8 mismatches), *Hepatozoon americanum* (16 mismatches) and *Toxoplasma gondii* (15 mismatches). The 6-FAM label is directly attached to the 3-terminal nucleotide of the fluorescein probe, and the LCRed-640 fluorescein label is added via a linker to the 5′-end of the LCRed-640 probe.

The 18S rRNA sequences for the available recognized *Theileria* spp. on GenBank and 4 other closely related protozoan species were obtained from GenBank: *T. orientalis* (HM538222), *T. buffeli* (HQ840967), *T. annulata* (KF429799), *T. sergenti* (EU083804), *T. luwenshuni* (JX469527), *T. velifera* (AF097993), *T. ovis* (AY508458), *T. parva* (L02366), *T. uilenbergi* (JF719835), *T. equi* (AB515310), *T. lestoquardi* (JQ917458), *T. separata* (AY260175), *T. capreoli* (AY726011), *T. cervi* (AY735119), *T. bicornis* (AF499604), *T. taurotragi* (L19082), *T. mutans* (FJ213585); *Babesia bovis* (KF928529), *B. divergens* (LK935835), *B. bigemia* (LK391709), *Hepatozoon americanum* (AF176836), *Cytauxzoon felis* (AY679105) and *Toxoplasma gondii* (L37415) (Figure 1). The Clustal Multiple Alignment Algorithm was used to identify a highly conserved region of the 18S rRNA gene common to all the above *Theileria* spp. but significantly different from the other protozoan species (Figure 1). The primers and probes we developed were situated within the conserved region and synthesized by Integrated DNA Technologies (Coralville, IA, USA). The *Theileria* FRET-qPCR we established amplifies a 149-bp target with the positions of primers and probes shown in Figure 1: forward primer: 5′-TAGTGACAAGAAATAACAATACGGGGCTT-3′; reverse primer: 5′-CAGCAGAAATTCAACTACGAGCTTTTTAACT-3′; anchor probe: 5′-CCAATTGATACTCTGGAAGAGGTTT-(6-FAM)-3′; reporter probe: 5′-(LCRed640)-AATTCCCATCATTCCAATTACAAGAC-phosphate-3′.

#### Thermal cycling

The *Theileria* FRET-PCR was performed in a LightCycler 480**®**II real-time PCR platform with 20 μl volumes comprising 10 μl reaction master mix and 10 μl of sample. Thermal cycling consisted of a 2 min denaturation step at 95°C followed by 18 high-stringency step-down thermal cycles, 40 low-stringency fluorescence acquisition cycles, and melting curve determination between 38°C and 80°C. The parameters for qPCR were 6 × 12 sec at 64°C, 8 sec at 72°C, 0 sec at 95°C; 9 × 12 sec at 62°C, 8 sec at 72°C, 0 sec at 95°C; 3 × 12 sec at 60°C, 8 sec at 72°C, 0 sec at 95°C; 40 × 8 sec at 54°C and fluorescence acquisition, 8 sec at 72°C, 0 sec at 95°C.

#### Specificity

PCR products were verified using electrophoresis (1.5% MetaPhor agarose gels), followed by purification with a QIAquick PCR Purification Kit (Qiagen, Valencia, CA, USA) and genomic sequencing (GenScript, Nanjing, Jiangsu, China). The sequencing data from randomly selected positive *Theileria* samples (n = 37) were compared with the existing *Theileria* sequences in the GenBank using BLAST. The specificity of the PCR was further verified with the amplification of *T. orientalis* rRNA-containing pIDTSMART cloning Vector (Integrated DNA Technologies, Coralville, IA, USA) and 100 DNA copies of the rRNA gene of *B. canis*, *H. americanum*, *C. felis* and *T. gondii* (kindly provided by the parasitological laboratory of Yangzhou University College of Veterinary Medicine).

#### Sensitivity

For use as quantitative standards, the PCR products of DNAs of 5 *Theileria* species (*T. orientalis*, *T. sergenti*, *T. buffeli*, *T. luwenshuni*, *T. ovis*) were gel purified using a QIAquick Gel Extraction Kit (Qiagen, Valencia, CA, USA). After using the estimated molecular mass of the rRNA gene and the Quanti-iT TM PicoGreen ® dsDNA Assay Kit (Invitrogen Corporation, Carlsbad, CA, USA) to calculate the molarity of the solution, dilutions were made to give solutions containing 10,000, 1,000, 100, 10, 1 gene copies per PCR reaction system. These dilutions, and further dilutions providing 2, 4, 6 and 8 gene copies per PCR reaction, were used to determine the minimal detection limit. The 10-fold dilutions were used as quantitative standards in the FRET-PCR surveys to enable standard curves to be developed for the calculation of the gene copy numbers in positive samples.

### Identification of *Theileria*spp. by PCR and sequencing

The amplicon of the pan-*Theileria* FRET-qPCR we established has a sequence which is highly conserved among the different *Theileria* species. To differentiate *Theileria* spp. in a positive reaction, we used a standard PCR to amplify a highly polymorphic region of the 18S rRNA gene (591–594 nucleotides for different *Theileria* spp.) and sequenced the products (GenScript, Nanjing, Jiangsu, China). For the PCR we designed a forward primer (5′-CCTGAGAAACGGCTACCACATCT-3′) that amplified all *Theileria* species and used a previously described reverse primer (5′-GGACTACGACGGTATCTGATCG-3′) that also amplified all species [31].

### Statistical analysis

Differences in positivity of *Theileria* spp. were analyzed by Chi-squared Test while numbers of copies of the *Theileria* 18S rRNA gene determined in the *Theileria* FRET-qPCR were log10-transformed and analyzed using the Student’s T-test. Differences of *P* < 0.05 were considered statistically significant.

## Results

### Development of the pan-*Theileria*FRET-PCR

Comparison of the sequences in the highly conserved region of the *Theileria* spp. we used showed the region is highly conserved, but is substantially different from those in closely related protozoan species (Figure 1). The two primers and two probes we chose for the pan-*Theileria* FRET-qPCR had 0–4 nucleotide mismatches with the *Theileria* spp. in GenBank, but had 25, 23, 22, 8, 16 and 15 mismatches with *B. bovis*, *B. divergens*, *B. bigemina*, *C. felis*, *H. americanum* and *T. gondii*, respectively (Figure 1). The specificity of the pan-*Theileria* FRET-PCR was further confirmed when it gave positive reactions with the *T. orientalis* control, but gave negative reactions with DNAs of *B. canis*, *C. felis*, *H. americanum* and *T. gondii*. The pan-*Theileria* FRET-qPCR had a specific melting curve (*T*_m_ 57.5°C) with *Theileria* spp. DNA. Using the gel-purified PCR products as quantitative standards, we determined the detection limit of the pan-*Theileria* FRET-qPCR was 2 copies of the *Theileria* 18S rRNA gene per reaction for *T. orientalis*, *T. sergenti* and *T. luwenshuni*, *T. buffeli* and *T. ovis*.

### Prevalence of *Theileria*spp. DNA in ruminants

**Figure 2:**
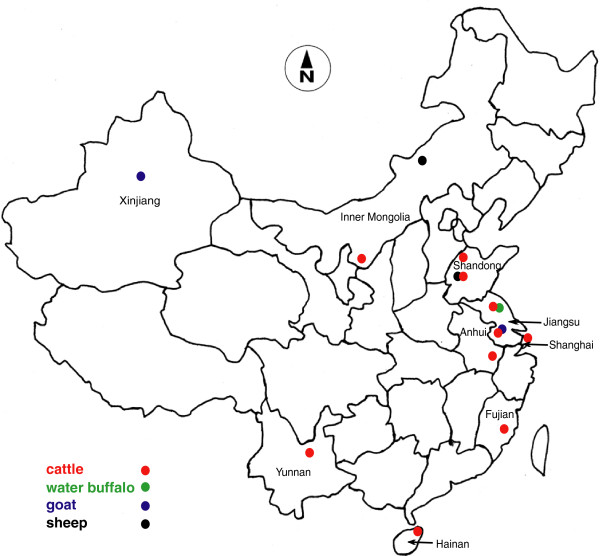
**Sites in China where samples ruminants were tested for** ***Theileria*** **spp. DNAs.** Dots of different colors represent sites where samples obtained from cattle, water buffalo, goat and sheep of nine provinces were tested by pan-*Theileria* FRET-qPCR in this study.

Animals positive for *Theileria* were found in each of the nine provinces sampled with several animals of each species being positive at each location, except in the case of water buffaloes which were all negative in the one site they were studied. The overall prevalences of *Theileria* spp. DNA in sheep (53.2%; 59/111) and goats (44.4%; 120/270) were significantly higher than in cattle (30.8%; 380/1,235) (two-tailed Chi-squared Test, *P* < 10^-4^). The pan-*Theileria* FRET-PCR showed that sheep had an average of 10^2.4^ copies of *Theileria* 18S rRNA/ml whole blood which was significantly lower than the 10^4.3^ copies in cattle and 10^5.8^ copies in goats (Student’s t Test, *P* < 10^-4^). While the prevalence of *Theileria* spp. DNA varied greatly from 3.5% (9/255) in Holsteins from Shanghai to 100% in Luxi cattle from Shandong (40/40) and Leiqiong cattle from Hainan (74/74), the prevalence did not differ significantly in sheep from Inner Mongolia and Shandong (Table 1, Figure 2).

Sequencing of 87 randomly selected amplicons (52 from cattle, 14 from goats and 21 from sheep) from *Theileria* DNA positive samples showed that *T. orientalis/ T. sergenti/T. buffeli* group [29] were present in cattle while *T. luwenshuni* was found in goats in Jiangsu province and *T. ovis* and *T. luwenshuni* in sheep from Inner Mongolia and Jiangsu province, respectively.

### Factors associated with the occurrence of theileriosis in cattle

When we analyzed factors that might be associated with the prevalence of *Theileria* spp. DNA in cattle we found that *Bos p. indicus* animals had significantly higher positivity (77.7% vs. 18.3%; *P* < 10^-4^) and copy number of the *Theileria* 18S rRNA gene (10^4.81^ vs. 10^3.73^ copies/per ml whole blood; *P* < 10^-4^) than *Bos p. taurus* animals. Similarly, *Bos p. indicus* cattle were more likely to be positive (65.2% vs. 13.7%; *P* < 10^-4^) and have higher copy numbers of the *Theileria* 18S rRNA gene (10^4.88^ vs. 10^3.00^ copies/per ml whole blood; *P* < 10^-4^) than *Bos p. taurus* animals. The cattle from southern China had significantly higher *Theileria* 18S rRNA gene copy numbers (10^4.39^ vs. 10^3.87^ copies/per ml whole blood; *P* = 0.02) than those from northern China but this difference in the prevalence was not significant (31.8% vs. 26.5%). In cattle from Yunnan province where gender information was available, female animals were more commonly positive (76.7% vs. 41.3%; *P* < 10^-4^) and copy numbers (10^5.02^ vs. 10^2.93^*Theileria*/per ml whole blood; *P* < 10^-4^) than males.

### Gene accession numbers

The *Theileria* rRNA nucleotide sequences obtained in this study that were not identical to existing entries in GenBank were deposited with the following gene accession numbers: KJ850933 and KJ850938 (*T. sergenti*); KJ850936 and KJ850940 (*T. buffeli*); KJ850934, KJ850937, KJ850943, KJ850939 and KJ850941 (*T. orientalis*); KJ850942 (*T. ovis*); KJ850935 and KM016463 (*T. luwenshuni*). The sequences obtained were very similar (0–4 nucleotide mismatches) to *Theileria* spp. sequences deposited by other laboratories in China, USA, France, Australia and Iran (Table 2).

**Table 2:** **Comparison of isolates identified in this study and similar sequences in GenBank by BLASTN**

Isolates identified in this study			Highly similar sequences in GenBank		
***Theileria***spp.	Gene accession #	Source/origin	Gene accession #	Source/origin	Mismatches
***T. orientalis***	KJ850934	Simmental cattle, Inner Mongolia	AP011948	Cattle, Shintoku of Japan	0/547
	KJ850939	Luxi cattle, Shandong	HM538220	Cattle, Suizhou of China	0/547
	KJ850937	Yunling cattle, Yunnan	AB520956	Cattle, New South Wales of Australia	2/491
	KJ850941	Yunling cattle, Yunnan	AB520955	Cattle, Raymond of Australia	0/509
	KJ850943	Holstein cattle, Jiangsu	AB520956	Cattle, New South Wales of Australia	0/549
***T. sergenti***	KJ850933	Australian Holstein cattle, Jiangsu	JQ723015	Cattle, Hunan of China	0/541
	KJ850938	Yunling cattle, Yunnan	JQ723015	Cattle, Hunan of China	0/492
***T. buffeli***	KJ850936	Leiqiong cattle, Hainan	HM538196	Cattle, Hubei of China;	1/508
	KJ850940	Wannan cattle, Anhui	AY661513	Cattle, USA	0/545
***T. ovis***	KJ850942	Wuranke sheep, Inner Mongolia	FJ603460	Sheep, Xinjiang of China;	0/529
***T. luwenshuni***	KJ850935	from Sishui-Fur sheep, Shandong	KC769996, JX469518, JF719831	Sheep, China	0/549
	KM016463	Yangtse River Delta White goat, Jiangsu	KC769997	Goat, Beijing of China	0/571

## Discussion

By systematically aligning the 18S rRNA sequences of representative *Theileria* spp. and other related protozoa, we identified a highly conserved region to place primers and probes for a pan-*Theileria* FRET-qPCR. The primers and probes we designed enabled us to amplify all our reference *Theileria* species with a detection limit of at least 2 *Theileria* 18S rRNA gene copies per PCR system. None of the other protozoan species tested gave reaction products. To the best of our knowledge, this is the first FRET-qPCR which specifically detects all *Theileria* species.

Our data indicate infections with *Theileria* spp. are very widespread and common in cattle in China. We found positive animals in each province where we tested and an overall average of 30.77% of animals being positive. This relatively high level of positivity is similar to that obtained in the only other molecular survey for *Theileria* in China which showed 13.46% being positive in the northeast [32]. Sequencing data showed cattle are infected with the *T. orientalis/T. sergenti/T. buffeli* group which are generally recognized to be benign species [33] although some strains might cause economic losses [34].

Although we sequenced relatively few positive amplicons, we found no evidence of the more pathogenic strains, *T. annulata* and *T. parva*, which is not unexpected in the case of *T. parva* which has only been reported from Africa [8]. *T. annulata*, however, has been reported in *H. asiaticum* in northwestern China [35]. Our failure to demonstrate the organism in our study might be because *T. annulata* has an uneven distribution in China.

Although we found no evidence of *Theileria* spp. in the 29 water buffaloes we sampled, He [36] reported 58/304 (19.1%) positive for *T. buffeli* in Hubei province, south China. More extensive studies are necessary to determine the epidemiology of theileriosis in water buffaloes.

We found both goats and sheep were infected with *T. luwenshuni* (Table 2) using the pan-*Theileria* FRET-PCR and sequencing. This is a highly pathogenic organism that is known to occur in China where it is transmitted by *Haemaphysalis qinghaiensis* and may cause significant economic losses. While PCR assays have been described which detect and differentiate *T. luwenshuni* from *T. uilenbergi* [37, 38], they do not enable detection of all *Theirleria* spp. in all species and are thus not as versatile as our pan-*Theileria* FRET-PCR. Yin *et al.* [37] found the prevalence of *T. luwenshuni* varied from 0% to 85% in 4 provinces and we also found a wide range of positive values (4.1% to 67.4%). Such variability is probably due to tick prevalence rates, geoclimatic factors and livestock management systems. Further research using sensitive detection methods will be important in determining the best mechanisms to control infections.

*T. ovis* was first identified in China in 2011 by PCR and sequencing and an infection rate of 78% was found in Xinjiang but no positive animals were found in twelve other provinces [26, 27, 39]. Our study confirms the presence of the organism in China although *T. ovis* is considered benign and economically unimportant as it might only cause signs in some animals which are stressed [40].

It is well recognized that exotic breeds are more susceptible to disease following infection with *Theileria* spp. than local stocks. Recent studies, however, have indicated that different breeds of animals might have similar susceptibilities to infections with *Theileria* spp. although some breeds are more capable of controlling the pathogenic effects of the organism [41].

Generally, *B. p. taurus* breeds are more susceptible to theileriosis than *B. p. indicus* breeds which might be associated with innate or immune mechanisms and/or general resistance to ticks. These factors have been mainly investigated with *T. parva* infections and thus it is interesting that our pan-*Theileria* FRET-PCR testing showed that in China it is the *B. p. indicus* breeds that are more likely to be positive than the *B. p. taurus* breeds and that the former generally had higher copy numbers indicating heavier infections. While our sample sizes were small and we cannot exclude the possibility of sample bias, our findings might be due to host genetic factors relating to infections with less pathogenic *Theileria* spp. or other factors such as differences in tick control and husbandry practices on different farms. Further studies taking these factors into account are needed to more precisely investigate the relationships between infections with benign *Theileria* spp. and the genetic background of the host.

## Conclusion

In summary, our study has described the development and testing of a FRET-PCR which can detect recognized *Theileria* spp. The pan-*Theileria* FRET-PCR should be a useful diagnostic tool as it will enable diagnostic laboratories to detect infections in all domestic species with a single test.
